# Splenic sarcoid reaction mimicking metastases in patients after uterine cancer surgery: a report of two cases

**DOI:** 10.1186/s40792-023-01753-1

**Published:** 2023-09-20

**Authors:** Kei Kitamura, Toshiro Ogura, Ryoichi Miyamoto, Hiroyuki Ishida, Shinichi Matsudaira, Amane Takahashi, Hiroaki Kanda, Takashi Fukuda

**Affiliations:** 1 Department of Gastroenterological Surgery, Saitama Cancer Center, 780 Komuro, Ina-Machi, Kita-Adachi-Gun, Saitama, 362-0806 Japan; 2 Department of Gastroenterological Surgery, Tokyo Medical University Ibaraki Medical Center, Ibaraki, Japan; 3 Department of Pathology, Saitama Cancer Center, Kita-Adachi-Gun, Saitama, Japan

**Keywords:** Sarcoid reaction, Sarcoidosis, Uterine cancer, Spleen, Splenectomy

## Abstract

**Background:**

Tumor-associated sarcoid reactions have been observed with various tumors; however, they have not been reported with uterine cancer. We present two cases of splenic sarcoid reactions that mimicked metastases a few years after uterine cancer surgery.

**Case presentation:**

Case 1 involved a 67-year-old female patient diagnosed with endometrial cancer (pT1aN0M0, pStage Ia, grade 1). The patient underwent open total abdominal hysterectomy and bilateral salpingo-oophorectomy with pelvic lymphadenectomy. Three years after the initial surgery, computed tomography (CT) and positron emission tomography CT showed multiple splenic masses with increasing numbers and sizes. Splenic metastases were diagnosed, and laparoscopic splenectomy was performed. The histopathological analysis revealed sarcoid reactions in the spleen. Case 2 involved a 47-year-old female patient diagnosed with endometrial cancer (pT1aN0M0, pStage Ia, grade 1). The patient underwent laparoscopic total abdominal hysterectomy and bilateral salpingo-oophorectomy with pelvic lymphadenectomy. Two years after the initial surgery, multiple splenic masses were observed. We performed laparoscopic splenectomy for the splenic metastases. Granuloma formations were identified in the splenic specimen and perisplenic lymph nodes that were removed simultaneously, resulting in a final diagnosis of sarcoid reaction. A review of the lymph nodes at the time of the previous uterine surgery revealed granuloma formation. Other than the presence of splenic masses, no findings suggestive of recurrence were observed in these cases. Uterine cancer and sarcoid reactions progressed without recurrence after splenectomy.

**Conclusions:**

To the best of our knowledge, this is the first report of the late development of splenic sarcoid reactions after uterine cancer surgery. Sarcoid reactions and metastases are difficult to diagnose based on preoperative imaging results. However, reviewing the specimen at the time of the initial resection, the number of lesions, and the clinical findings (other than imaging findings) may aid in the determination of the correct diagnosis.

## Background

Sarcoidosis is a multisystem granulomatous disease of unknown etiology that most frequently causes inflammation in the pulmonary system, skin, or lymph nodes. Sarcoid or sarcoid-like reactions are histologic changes characterized by non-caseating epithelioid cell granulomas in patients without systemic sarcoidosis [1, 2]. Sarcoid reactions have been associated with various tumors [1, 3, 4], including lymphoma, lung cancer [5–9], breast cancer [10–12], esophageal cancer [13], gastric cancer [14], colorectal cancer [15–19], biliary cancer [20, 21], pancreatic cancer [22], melanoma [23, 24], renal cell carcinoma [25–30], and bone cancer [31]. Sarcoidosis-like granulomas are predominantly found in the lymph nodes and stroma adjacent to tumors; however, involvement in the organs, such as the bone, skin, and spleen, is infrequent. Evidence-based clinical management and treatment strategies for cancer-associated sarcoid reactions remain poorly understood. Furthermore, the preoperative differential diagnosis based on malignant tumors, including metastasis from primary cancer, is known to be difficult [32].

We present two cases of sarcoidosis-like granulomas occurring in the spleen and lymph node after surgery for uterine cancer. These cases highlight the challenge of preoperatively differentiating sarcoidosis-like granulomas from splenic metastases. Additionally, we review the relevant literature regarding cancer-associated sarcoid reactions and discuss their clinical and therapeutic management and radiological features.

## Case presentation

### Case 1

**Fig. 1:**
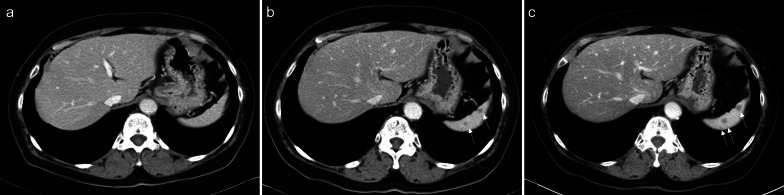
Time series findings in the spleen of case 1 obtained using CT. **a** No obvious splenic tumor is observed before the initial surgery. **b** CT images show several splenic tumors 3 years after the initial surgery. **c** Four years after the initial surgery, the splenic tumors are increased. *CT* computed tomography

**Fig. 2:**
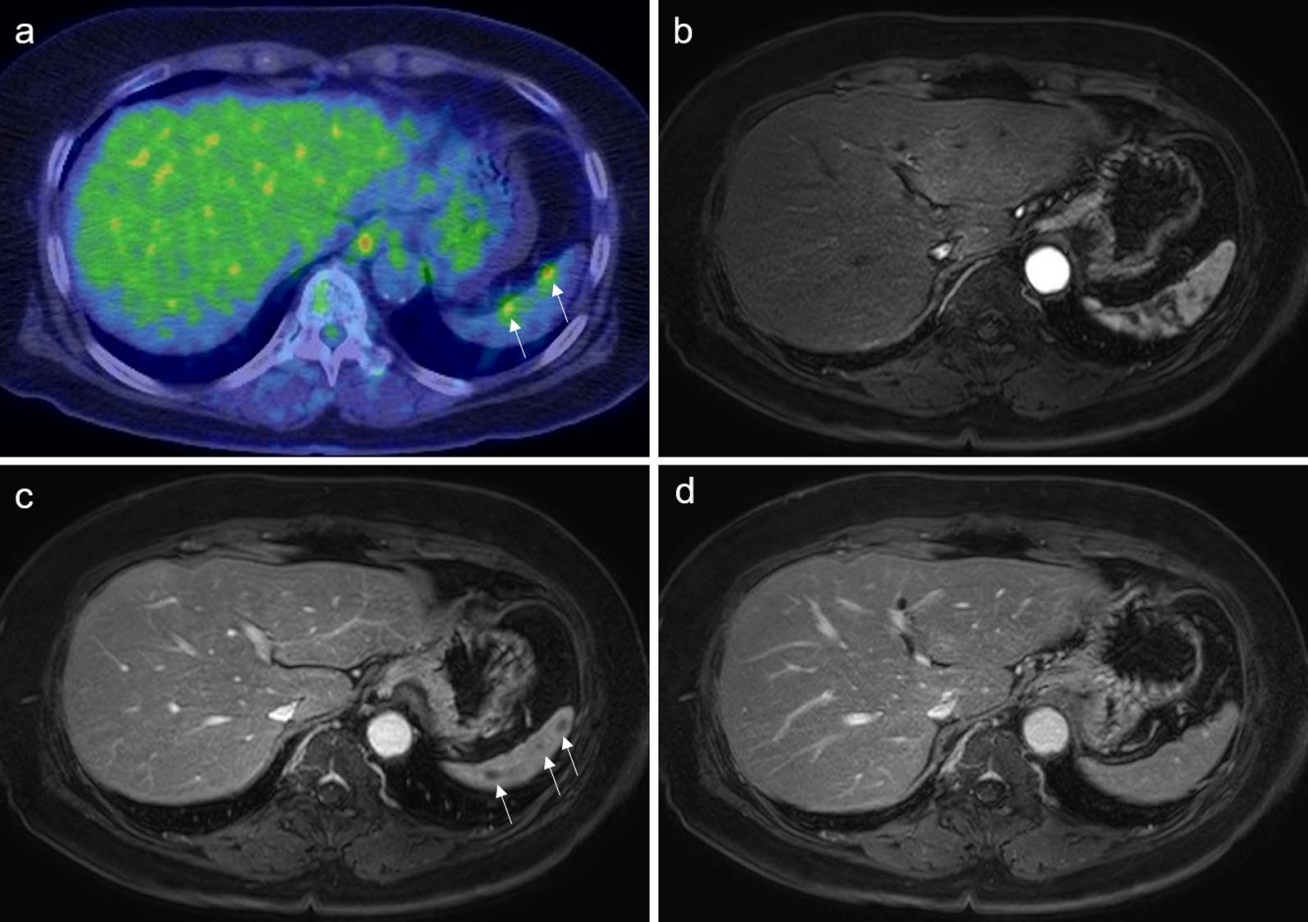
PET/CT and MRI findings of case 1. **a** PET/CT shows little 18F-fluorodeoxyglucose uptake at the site of the mass (maximum standardized uptake value: 4.11–4.95) in case 1. **b** MRI shows speckled contrast within the spleen during the early phase. **c** MRI during the late phase shows several ring-enhanced nodules in the spleen. **d** MRI shows no obvious nodules during the delayed phase. *PET/CT* positron emission tomography/computed tomography, *MRI* magnetic resonance imaging

A 67-year-old female patient was diagnosed with endometrial cancer (pT1aN0M0, pStage Ia according to the eighth edition of the Union for International Cancer Control (UICC), grade 1 tumor differentiation). The patient underwent open total abdominal hysterectomy (TAH) and bilateral salpingo-oophorectomy (BSO) with pelvic lymphadenectomy (PLA). Contrast-enhanced computed tomography (CT) before the initial surgery revealed no obvious splenic tumors (Fig. 1a). Three years after the initial resection, CT revealed five small low-density lesions with diameters of 3–6 mm (Fig. 1b). Four years after the initial resection, the size and number of splenic tumors increased (Fig. 1c). Some 18F-fluorodeoxyglucose (FDG) uptake in the mass [maximum standardized uptake value (SUV): 4.11–4.95] was observed using FDG positron emission tomography (PET)/CT (Fig. 2a). Magnetic resonance imaging (MRI) showed speckled contrast within the spleen during the early phase (Fig. 2b) and enhanced nodules similar to those observed with CT or PET/CT (Fig. 2c). However, no obvious nodules were observed during the equilibrium phase (Fig. 2d) on T1-weighted, T2-weighted, diffusion-weighted, or apparent diffusion coefficient map images obtained with MRI. MRI did not show any other obvious malignant findings; however, both the number and size of the splenic masses increased monthly (Fig. 1), and multiple uterine cancer metastases were suspected. The patient underwent laparoscopic splenectomy. The postoperative course was uneventful, and the patient was discharged on the 6th postoperative day.

**Fig. 3:**
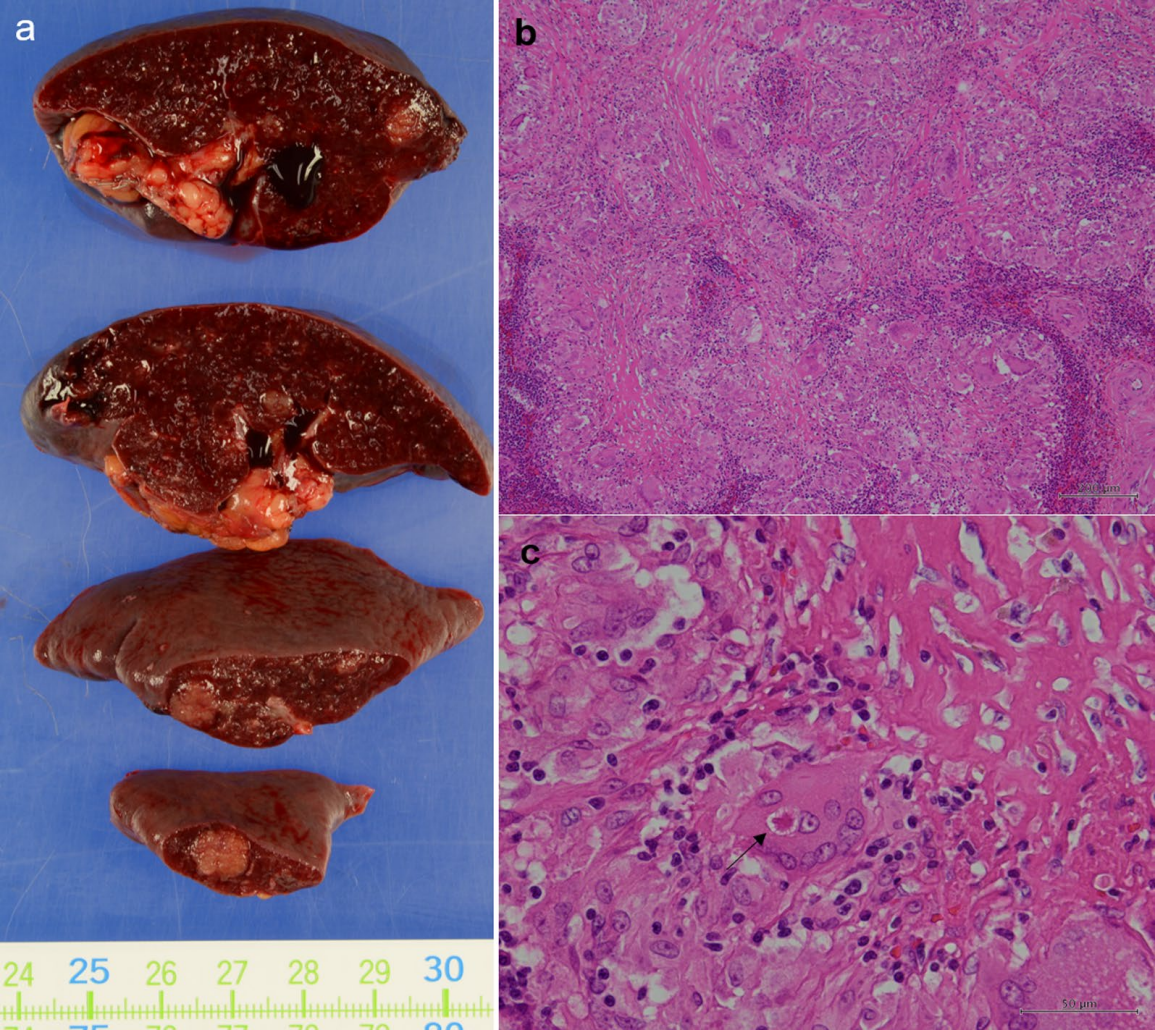
Histopathological findings of case 1. **a** Macroscopic view of the splenic mass. **b** Hematoxylin and eosin staining of splenic tumors with non-caseating granulomas. **c** Langerhans giant cells and asteroid body (arrow)

Histopathological examination revealed multiple 3- to 6-mm epithelioid granulomas in the spleen (Fig. 3a). These nodules exhibited Langerhans giant cells and asteroid bodies. Dry necrosis was not prominent (Fig. 3b, c), and Ziehl–Neelsen staining yielded negative results. The preoperative serum soluble interleukin 2 receptor (sIL-2R) level was 408 U/ml. Neither clinical nor radiological evidence of respiratory, ocular, cutaneous, cardiac, or neurologic lesions suggestive of sarcoidosis was observed. These findings suggested a splenic sarcoid reaction. The specimen did not contain any lymph nodes. The lymph nodes removed during uterine cancer surgery were retrospectively examined; however, no granulomas were observed.

### Case 2

**Fig. 4:**
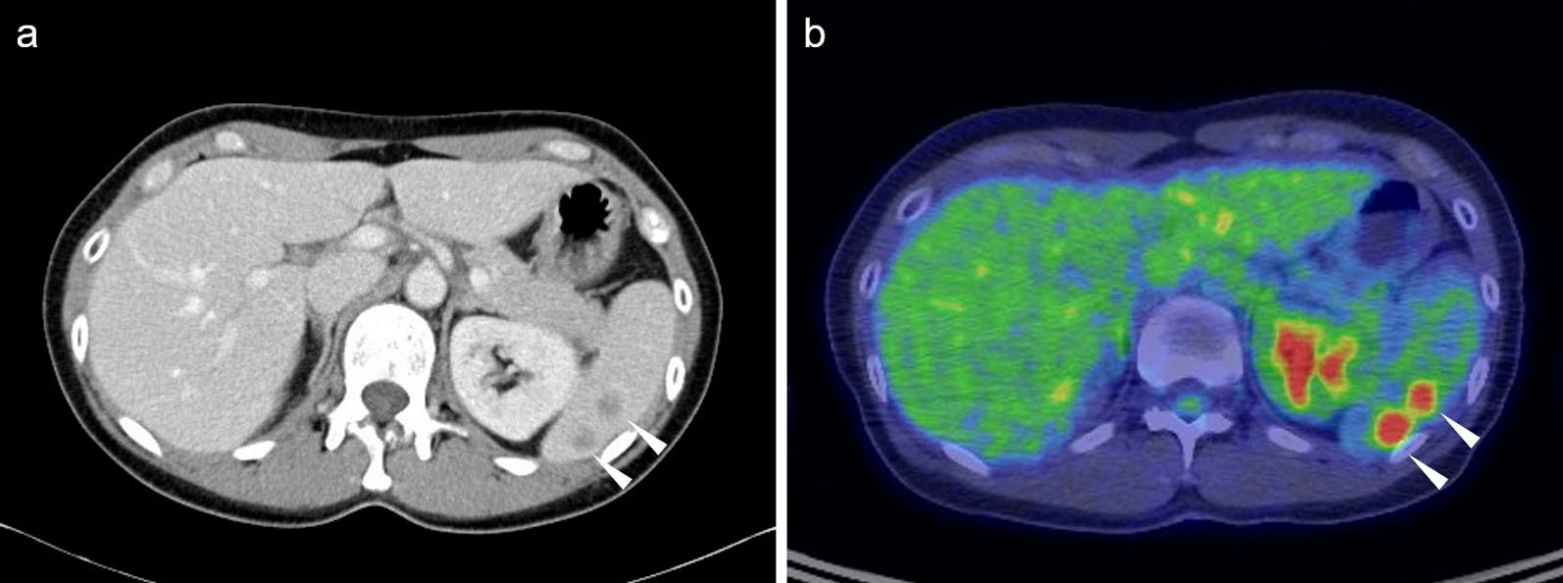
CT and PET/CT findings of case 2. **a** CT image shows small low-density lesions. **b** PET/CT shows high 18F-fluorodeoxyglucose uptake at the site of the mass (maximum standardized uptake value: 7.07–8.51) in case 2. Arrowheads indicate splenic tumors. *CT* computed tomography, *PET/CT* positron emission tomography/computed tomography

**Fig. 5:**
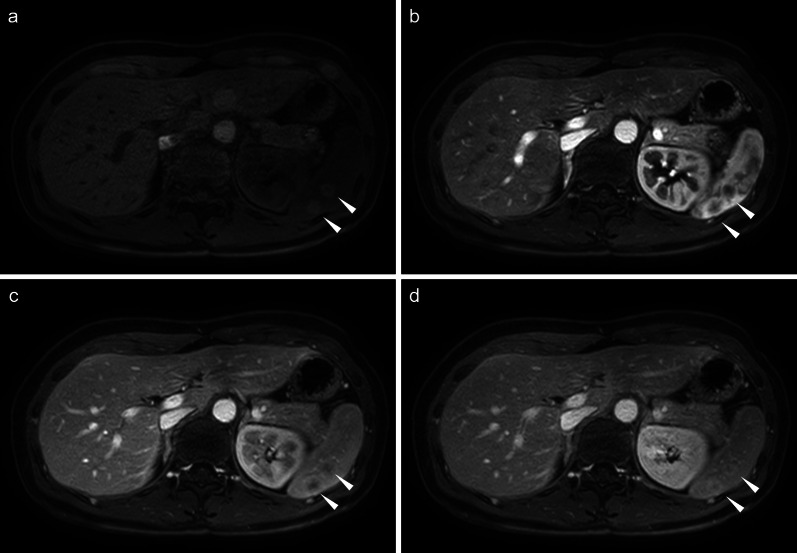
Magnetic resonance imaging findings of case 2. **a** Multiple ring-shaped, high-signal nodules are observed in the spleen on T1-weighted images. **b** Early phase, **c** late phase, and **d** delayed phase of the dynamic study. Arrowheads indicate splenic tumors

A 47-year-old female patient underwent laparoscopic TAH, BSO, and PLA for endometrial cancer (pT1aN0M0, pStage Ia according to the eighth edition of the UICC, grade 1 differentiation). Two years after the primary surgery, CT revealed four small low-density lesions (Fig. 4a). PET/CT images also showed high FDG uptake at the site of the mass (maximum SUV: 7.07–8.51) (Fig. 4b). MRI T1-weighted images revealed multiple ring-shaped, high-signal nodules in the spleen (Fig. 5a). Pale enhancement effects in the same areas were observed during dynamic studies (Fig. 5b–d). Uterine cancer metastases, lymphoma, abscess, and other benign and malignant tumors were included in the differential diagnosis. The patient underwent laparoscopic splenectomy and was discharged on the 6th postoperative day without any postoperative complications.

**Fig. 6:**
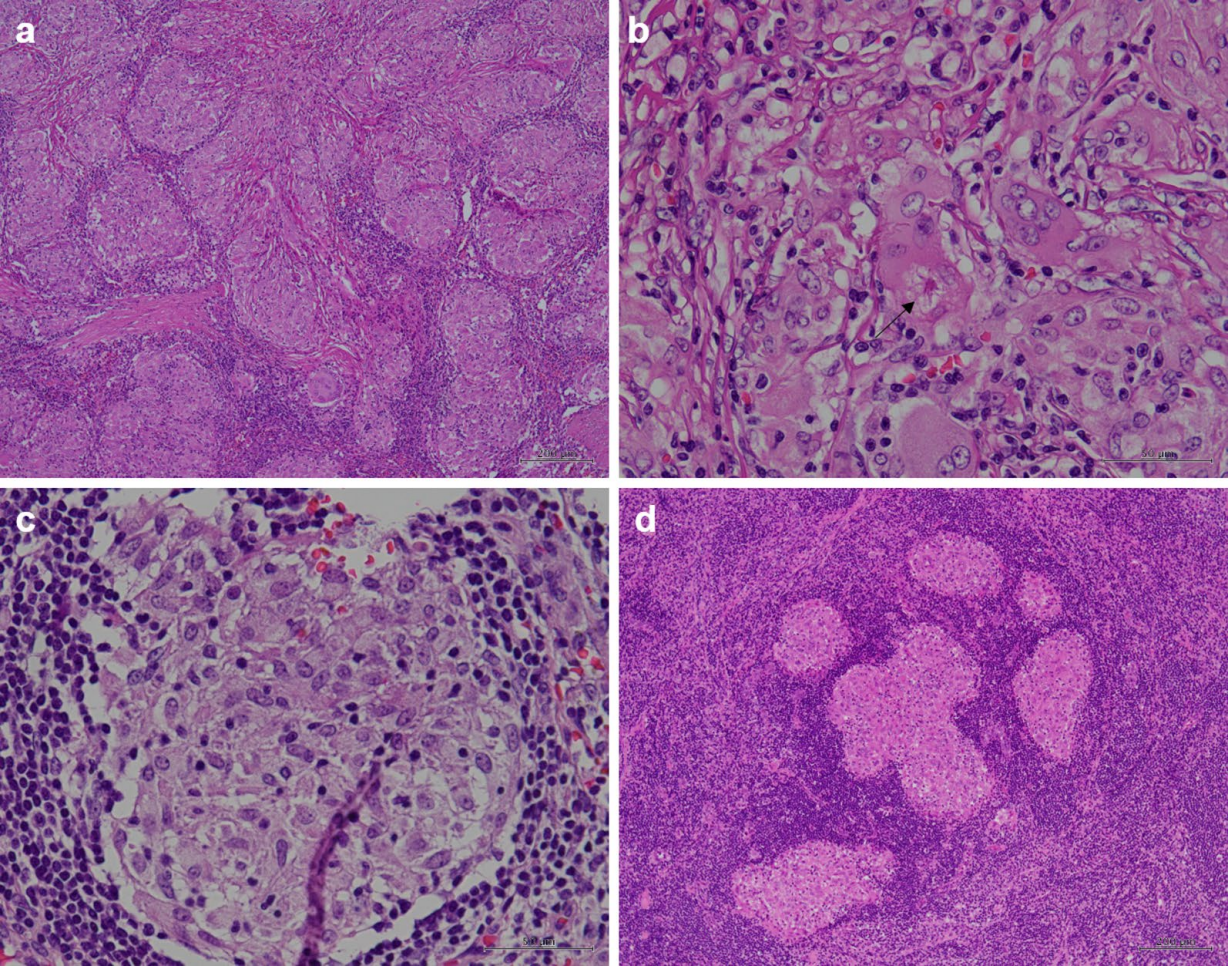
Histopathological findings of case 2. **a** HE staining of the spleen with multiple granulomas. **b** Langerhans giant cells and asteroid body (arrow). **c** HE staining of the splenic hilar lymph node showing granuloma formation. **d** Granuloma formation in the lymph nodes dissected at the time of the initial resection. *HE* hematoxylin and eosin

The histopathological diagnosis revealed multiple epithelioid cell granulomas with diameters of up to 15.2 mm in the spleen (Fig. 6a, b). Additionally, a 0.3-mm epithelioid cell granuloma was identified in the splenic hilar lymph node (Fig. 6c). A retrospective histopathological examination of the lymph node specimen, which was dissected at the time of the initial surgery, revealed limited granuloma formation in left external iliac lymph nodes (Fig. 6d). The sIL-2R level was not elevated preoperatively (383 U/ml) or postoperatively (308 U/ml). The postoperative serum angiotensin-converting enzyme level was 16.5 U/l. There were no clinical or imaging findings suggestive of sarcoidosis.

## Discussion

The present report describes two relatively rare cases of splenic sarcoid reactions after uterine cancer surgery. Both cases involved early-stage uterine cancers. Interestingly, they both exhibited disease progression without recurrence despite the absence of preoperative or postoperative treatment. To the best of our knowledge, no reports of splenic sarcoid reactions after uterine cancer resection have been published.

Sarcoid reaction involves the same histologic changes as sarcoidosis, but without the systemic disease of sarcoidosis [33]. In both cases, the laboratory and imaging findings were not suggestive of sarcoidosis, suggesting sarcoid reaction secondary to tumor resection. Several reports have documented the association between sarcoid reactions and various types of malignant tumors. The factors that cause malignancies to elicit sarcoid reactions are not clearly understood; however, many cases involve therapeutic agents, including immunosuppressive drugs [7–9, 23, 24]. Therefore, we summarized the reports of sarcoid reactions with a history of surgery over the past decade, including information regarding adjuvant drug use (Table 1). We excluded possible cases of sarcoidosis. For cases of drug-induced sarcoid reactions, rapid improvement has been reported after drug discontinuation [7, 24, 34, 35]. However, in some cases, the sarcoid reactions persist for more than 1 year after the drug is discontinued [34, 36]. Sarcoid reactions have been reported to improve with steroid therapy [7, 35]. However, for cases of non-drug-induced sarcoid reactions, the response to drug discontinuation cannot be confirmed and is more difficult to differentiate based on imaging alone. Thus, a biopsy or diagnostic treatment is often required. No cases of non-drug-induced sarcoid reactions in which the reaction could be distinguished from metastasis based solely on radiological findings have been identified. Only one case of drug-induced sarcoid reaction could be diagnosed as sarcoid reaction by radiological findings after the drug was withheld [24]. Most sarcoid reactions are discovered at the time of primary tumor resection [5, 6, 8, 9, 13, 16, 18, 20–22, 26, 27, 29, 30]; however, some (both drug-induced and non-drug-induced sarcoid reactions) are discovered 1 month to 5 years after surgery [10, 14, 15, 17, 19, 23, 25, 28, 31]. Granuloma formation is often found in lymph nodes, whereas spleen sarcoid reactions are more frequently found with non-drug-induced sarcoidosis. Splenic biopsy is difficult because of the high risk of hemorrhage. Therefore, preoperative differentiation between splenic sarcoid reactions and metastatic recurrence can be challenging. Laparoscopic splenectomy can be performed safely and with minimal invasiveness, even when the initial surgery is laparotomy, as demonstrated by case 1 of the current report. This approach can reduce the physical burden on the patient.

**Table 1:** Summary of patients who underwent surgery for solid tumors with sarcoid reactions reported between 2012 and 2022, including the present cases

Patient	Author	Year	Age	Sex	Primary tumor	TNM stage	Adjuvant therapy for primary cancer	Tumor recurrence	Location of sarcoid reaction	Solitary or multiple	Time of sarcoid reaction after surgery
1	Tao [5]	2012	59	F	Lung cancer	N/A	None	No (1 year)	Lung parenchyma and lymph nodes	Multiple	Simultaneous
2	Fong [21]	2012	59	M	Cholangiocarcinoma	N/A	None	No (9 months)	Spleen	Multiple	Simultaneous
3	Fong [21]	2012	68	F	IPMN with carcinoma in situ	N/A	None	No (9 months)	Spleen, liver, bone	Multiple	Simultaneous
4	Quellet [30]	2012	62	M	Renal cancer	N/A	None	No (30 months)	Normal renal parenchyma	Multiple	Simultaneous
5	Craun [31]	2012	34	F	Bone cancer	N/A	Radiation therapy	Yes (less than 1 year)	Mediastinal, hilar, right axillary, and right neck lymph nodes	Multiple	4 years
6	Mastroroberto [22]	2012	52	M	Neuroendocrine tumor of the pancreas	N/A	None	N/A	Lymph nodes	Multiple	Simultaneous
7	Burhan [29]	2013	62	M	Renal cancer	N/A	None	N/A	Nephrectomy specimen	Solitary	Simultaneous
8	Fiorelli [19]	2014	67	F	Colorectal cancer	N/A	6 cycles of adjuvant	No	Paratracheal lymph node	Multiple	5 years
9	Kawasaki [20]	2014	73	F	Gallbladder cancer	T1bN0M0, stage IA	None	No (4 years)	Regional lymph node	Multiple	Simultaneous
10	Ritterhouse [27]	2014	34	F	Renal cancer	T1b	None	No (22 months)	Tumor stroma	Multiple	Simultaneous
11	Madden [28]	2014	41	M	Renal cancer	T3aNxMx	None	No (15 months)	Axial and proximal appendicular skeleton	Multiple	2 years
12	Khatua [26]	2015	42	F	Renal cancer	N/A	None	N/A	Intratumoral epithelioid granuloma	Multiple	Simultaneous
13	Rubinstein [11]	2015	76	F	Breast cancer	N/A	Radiation and tamoxifen	No (30 months)	Right preauricular and right upper cervical sentinel lymph nodes	Multiple	N/A
14	Shima [17]	2016	76	F	Colorectal cancer	T3N2M0	S-1, FOLFOX	Yes (4 months)	Spleen	Solitary	20 months
15	Diaz del Arco [18]	2016	55	M	Colorectal cancer	T3N1M0	None	N/A	Within the stroma adjacent to the carcinoma	Multiple	Simultaneous
16	Pascual-Camps [6]	2017	60	M	Lung cancer	T2bN0M0, stage IIA	None	Yes	Lymph nodes and tumor, ocular	Multiple	Simultaneous
17	De Gregorio [16]	2018	32	F	Colorectal cancer	T3N1M0	FOLFOX + cetuximab	No	Bilateral mediastinal and hilar lymph nodes	Multiple	Simultaneous
18	Iftikhar [25]	2019	45	F	Renal cancer	N/A	None	N/A	Mediastinal and bilateral hilar nodes	Multiple	1 year
19	Garanzini [24]	2019	66	F	Melanoma	T3N1aM0	Ipilimumab	No (5 years)	Lung and spleen	Multiple	20 months
20	Yousuf [7]	2020	62	F	Lung cancer	T1cN0M0	Pembrolizumab	N/A	Mediastinal and right hilar and subcarinal lymphadenopathy	Multiple	N/A
21	Irie [13]	2020	52	F	Esophageal cancer	T1N0M0, stage IA	5-Fluorouracil, docetaxel plus cisplatin	No (4 years)	Regional lymph node	Multiple	Simultaneous
22	Okada [14]	2020	82	M	Gastric cancer	T3N0M0, stage IIA	None	No (24 months)	Spleen	Solitary	6 months
23	Aedma [15]	2020	54	M	Colorectal cancer	IVB	FOLFOX + bevacizumab	No (18 months)	Hepatosplenic lesions, mediastinal and bilateral hilar lymph nodes	Multiple	6 months
24	Frohlich [23]	2020	57	M	Melanoma	T4N1M0, stage IIIc	Either ipilimumab or nivolumab	No (40 weeks)	Bilateral hilar, prevascular, and right lower paratracheal lymphadenopathy	Multiple	14 weeks
25	Shi [8]	2021	68	F	Lung cancer	T4N0M0, stage IIIA	Pembrolizumab	No	Lung and mediastinal lymph node	Multiple	Simultaneous
26	Jeong [10]	2021	58	F	Breast cancer	T1aN0M0	Cytoxan, methotrexate, and fluorouracil	N/A	Bilateral supraclavicular, bilateral mediastinal, and intraperitoneal areas	Multiple	40 months
27	Zhao [9]	2022	54	F	Lung cancer	T2N0M0, stage IB	Nivolumab	N/A	Lymph nodes	Multiple	Simultaneous
28	Present case 1		67	F	Uterine cancer	T1aN0M0, stage IA	None	No (4 years)	Spleen	Multiple	3 years
28	Present case 2		47	F	Uterine cancer	T1aN0M0, stage IA	None	No (5 years)	Spleen and lymph node	Multiple	2 years

*F* female, *FOLFOX* folinic acid/5-fluorouracil/oxaliplatin, *IPMN* intraductal papillary mucinous neoplasms, *M* male, *N/A* not available, *S-1* tegafur/gimeracil/oteracil potassium

As reported in the previous literature regarding splenic sarcoidosis, the contrast effect on CT and accumulation on PET/CT made differentiation from metastasis difficult [17, 21]. The images obtained with MRI were diverse and differed between cases 1 and 2, as observed during various studies reported in the literature [24]. In case 1, the lesions were indistinct on T1- and T2-weighted images and only appeared as a nodular low-signal area during the late phase of the dynamic study (Fig. 2b–d). In case 2, the lesions appeared as a faintly ring-shaped high-signal area on T1-weighted images (Fig. 5a) and during the dynamic study (Fig. 5b–d). In the previous literature, the lesions appeared feeble nodules on T1-weighted images, similar to those of case 2; however, contrast enhancement was not observed [24]. These findings were not compared or contrasted with similar findings in the previous literature. The definitive diagnosis of sarcoid reactions using current diagnostic imaging modalities is challenging.

Isolated splenic metastasis of endometrioid cancer is also rare, with only 19 cases reported in the literature [37, 38]. Previous studies have reported that splenic metastases are often identified as solitary lesions [37, 38]; however, two lesions have been occasionally observed [38]. No reports have documented more than three lesions in the spleen, as observed in the present cases. The number of intrasplenic lesions may help differentiate sarcoid reactions from metastases. In cases of low-grade T1a early-stage uterine cancer with a low risk of recurrence [39] and no findings other than splenic lesions to suggest recurrence, such as our cases, sarcoid reaction should be suspected. In cases of iatrogenic splenic sarcoidosis, granuloma formation may be observed in the initial resected specimen [14]. In case 2, but not in case 1, limited granuloma formation was observed in the lymph nodes of the initial resection specimen (Fig. 6d). Sarcoid reactions may not be initially considered when preoperative diagnosis of the tumor or sarcoid reaction using imaging is difficult; however, a review of the initial specimen for the presence of granuloma formation may help with their identification. The sarcoid reaction is thought to be involved in the T cell-type immune response [4] and is not associated with overall or recurrence-free survival [3]. There have been no reports of sarcoid reactions leading to sarcoidosis. A sarcoid reaction is not considered an indication for treatment. Therefore, careful follow-up is an option when a sarcoid reaction is suspected to be more likely than metastasis. A needle biopsy of splenic lesions is also considered, but this is not aggressively recommended because of the high risk of hemorrhage.

This research study describes a single-center experience. There is little previous literature available for comparison because of the uniqueness of the cases, thus limiting the generalizability of the results.

## Conclusions

This report describes two cases of splenic sarcoid reactions in the spleen after endometrial cancer surgery. A histological examination is standard for the diagnosis of sarcoid reactions because preoperative imaging differentiation is difficult. A review of the specimen at the time of the initial resection, the number of lesions, and the clinical findings other than imaging findings may help avoid additional surgery. Future research should aim to provide further clarification of the underlying causes of sarcoid reactions and explore effective methods for differentiating them from preoperative imaging or physical examination findings.

## Data Availability

Not applicable.

## Abbreviations

BSO: Bilateral salpingo-oophorectomy
CT: Computed tomography
FDG: 18F-fluorodeoxyglucose
MRI: Magnetic resonance imaging
PET: Positron emission tomography
PLA: Pelvic lymphadenectomy
sIL-2R: Soluble interleukin 2 receptor
SUV: Standardized uptake value
TAH: Total abdominal hysterectomy
UICC: Union for International Cancer Control
